# Oolonghomobisflavans from *Camellia sinensis* increase *Caenorhabditis elegans* lifespan and healthspan

**DOI:** 10.1007/s11357-021-00462-7

**Published:** 2021-10-12

**Authors:** Chatrawee Duangjan, Sean P. Curran

**Affiliations:** 1 Leonard Davis School of Gerontology, University of Southern California, Los Angeles, USA; 2 Molecular and Computational Biology, Dornsife College of Letters, Arts, and Science, University of Southern California, Los Angeles, USA

**Keywords:** *C. elegans*, Lifespan, Healthspan, Oolong tea, Natural products

## Abstract

Tea polyphenols are widely considered as excellent antioxidant agents which can contribute to human health and longevity. However, the identification of the active biomolecules in complex tea extracts that promote health and longevity are not fully known. Here we used the nematode *Caenorhabditis elegans* to analyze the health benefits and longevity effects of *Camellia sinensis* oolong tea extracts (QFT, NFT, and CFT) and oolonghomobisflavan A and oolonghomobisflavan B, which are present in oolong tea extracts. Our results showed that oolong tea extracts and oolonghomobisflavans prolong lifespan and improved healthspan by curtailing the age-related decline in muscle activity and the accumulation of age pigment (lipofuscin). We found that the lifespan and healthspan promoting effects of oolong tea extracts and oolonghomobisflavans were positively correlated with the stress resistance via DAF-16/FOXO transcription factor. Furthermore, oolong tea extracts and oolonghomobisflavans displayed protective effects against Aβ- and polyQ-induced neuro/proteotoxicity. Overall, our study provides new evidence to support the health benefits of oolong tea and importantly identify oolonghomobisflavans as potent bioactive molecules that promote health when supplemented with a normal diet. As such, oolonghomobisflavans represent a valuable new class of compounds that promote healthy aging.

**Supplementary Information:**

The online version contains supplementary material available at 10.1007/s11357-021-00462-7.

## Introduction

Genetics, lifestyle, and the environment affect human health over an organism’s lifespan. Age-related degenerative diseases including cancer, cardiovascular, and neurodegenerative diseases are major threats to human health [1, 2]. Accumulating evidence has been reported that oxidative stress is a major risk factor for age-related diseases [3–12]. An imbalance of reactive oxygen species (ROS) levels in an organism, and exposure to oxidative stress-promoting conditions, can cause oxidative damage to macromolecules (lipids, DNA, and proteins) which can accelerate age-related decline in function [3]. As such, strategies to protect against the effects of ROS are needed to promote health and healthy aging.

*Caenorhabditis elegans* is an established model organism to study aging and age-related disorders [13]. More importantly, *C. elegans* shares similar physiological traits with humans, including reduced physiological indexes with age [2, 14], which are regulated by conserved molecular pathways [2, 15]. The insulin/IGF-1 signaling (IIS) is a well‐known and evolutionarily conserved pathway that controls longevity via the forkhead box O (FoxO) transcription factor and its downstream targets [2, 15]. In *C. elegans*, the FoxO transcription factor DAF-16 plays an essential role in stress resistance, longevity, metabolism, and development [2, 16]. Recent studies have reported that similar to humans, the lifespan and healthspan of *C. elegans* are associated with stress tolerance and stress resistance [2, 9, 17]. Importantly, pharmacological treatment with antioxidants such as resveratrol [2, 18], anthocyanin [19], and epicatechin [20] have been demonstrated to improve lifespan and increase stress resistance, in part, by modulating DAF-16/FOXO signaling.

Although aging is an irreversible process, the rate of aging and the occurrence of age-related conditions can be delayed. Recently, the consumption of natural products has received considerable attention as a facile way to reduce the risk of age-related degenerative diseases [21]. For example, the health benefits of some fruits and vegetables are attributed to phytochemicals, especially polyphenols and other antioxidant compounds [22], although more research is needed to assess causality.

Diet is one of the most variable aspect of life between individuals and may account for a significant portion of the non-genetic effectors of lifespan and healthspan [11, 12, 23–35]. Tea, derived from the leaves of the evergreen plant *Camellia sinensis*, has attracted significant attention worldwide for its antioxidant and health benefits [36, 37]. *C. sinensis* tea contains several bioactive components such as polyphenols, pigments, polysaccharides, alkaloids, free amino acids, and saponins [36, 37]. Polyphenols (e.g., catechins and epigallocatechin gallate (EGCG)) are one of the major bioactive components in tea and possess multiple health-promoting activities including antioxidation [38], anti-inflammation [39], and have been associated with reduced severity of multiple age-related conditions like cancer [40], diabetes [41], and obesity [42]. Generally, teas can be classified into three major types depending on the production process: green tea (non-fermented), oolong tea (semi-fermented), and black tea (fully-fermented) [43]. The fermentation process is mediated by oxidative enzymes found in the leaves including polyphenol oxidase and peroxidase that result in the characteristic colors and distinct flavors of each type of oolong tea and black tea [43]. Oolong tea, derived from *C. sinensis* is a popular traditional Chinese tea in south China [44]. The pharmacology of tea has been intensively studied [36] and includes different preparations of tea, including Qing flavor Tieguanyin oolong tea (QFT), Nong flavor Tieguanyin oolong tea (NFT), and Chen flavor Tieguanyin oolong tea (CFT), which differ in the degree of fermentation. QFT, NFT, and CFT were fermented for 1 year (%), 2 years (%), and 10 years (%), respectively. The polyphenols found in oolong tea include epigallocatechin, epigallocatechin gallate (EGCG), epicatechin, and epicatechin gallate [45]. In addition, oolonghomobisflavan A (OFA) and oolonghomobisflavan B (OFB), are structurally distinct dimers of EGCG, are isolable from oolong tea but not from green tea or black tea [46]. Although several studies have been performed that describe the effects of tea polyphenols from green tea and black tea, the beneficial effects of oolong tea and its compounds are less well described. As such, here we investigate the healthspan and lifespan benefits of oolong tea and its specific compounds (OFA and OFB) in the *C. elegans* model.

## Results

### Oolong tea extracts increase lifespan in *C. elegans*

**Fig. 1:**
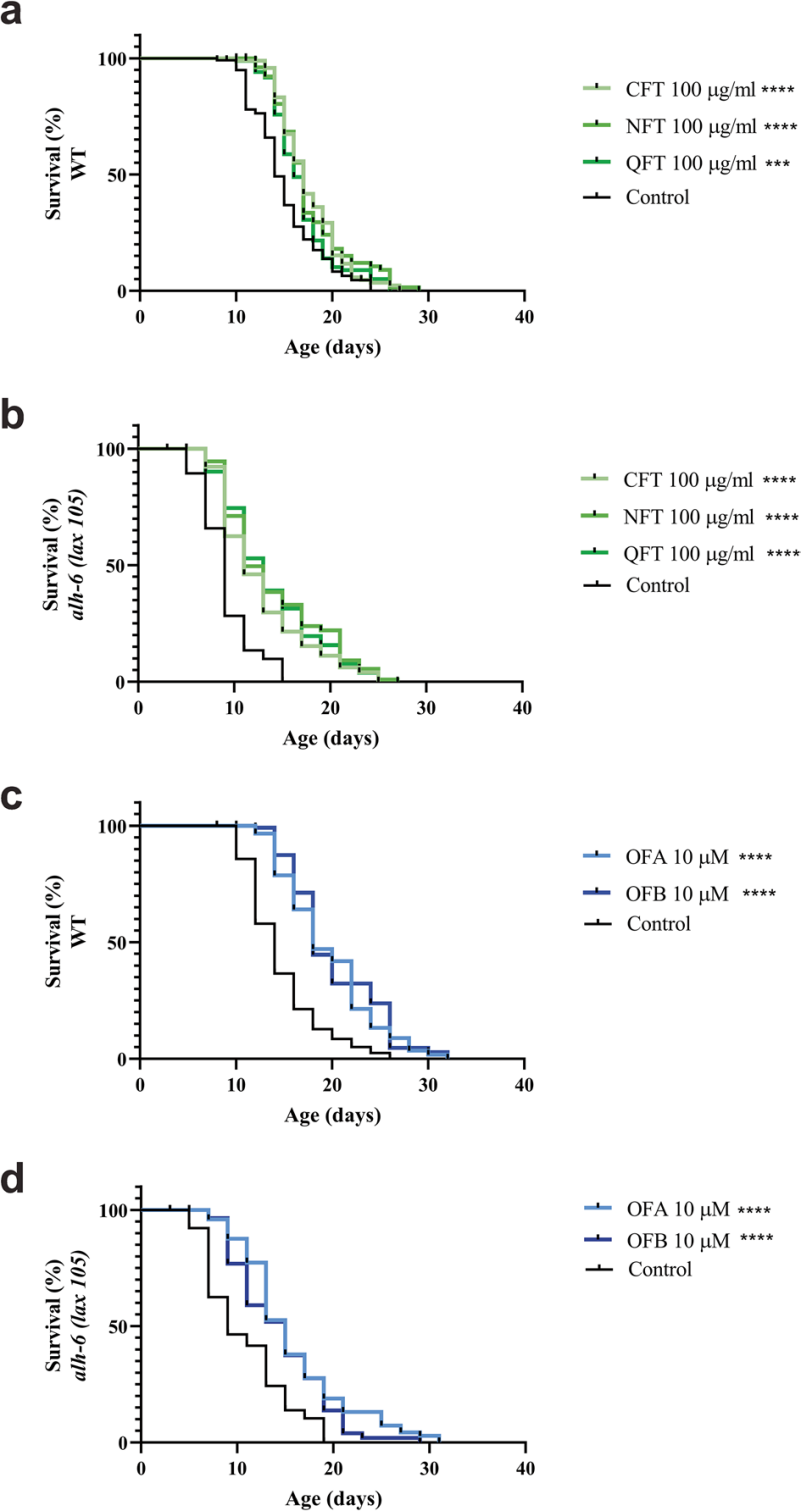
Effects of oolong tea extracts on lifespan. Survival curves of wild-type (N2) (**a**, **c**) and *alh-6(lax105)* (**b**, **d**) worms at 20 °C on the plate treated with oolong tea (QFT, NFT, CFT, OFA, OFB). *p < 0.05, **p < 0.01, ***p < 0.001, and ****p < 0.0001, compared to the mock-treated control by one-way ANOVA following log-rank test

We first tested the effect of *C. sinensis* oolong tea extracts on the lifespan of *C. elegans*. We treated wild-type worms with three types of oolong tea extracts (QFT, NFT, and CFT), which resulted in a dose dependent increase in mean lifespan relative to mock-treated controls (Fig. 1a, Table S1). We observed the most significant effect on lifespan from treatment with oolong tea extracts treatment at concentrations at 100 μg/ml and higher (Fig. S1a, c, e, Table S1), which increased the mean lifespan by approximately 10–20%, depending on the oolong tea extract used (Fig. 1a, Table S1). Because the mean lifespan did not further increase at 200 μg/ml, we used a concentration of 100 μg/ml for each oolong tea extracts in all subsequent experiments (Table S1).

Previously, we documented a premature aging phenotype in animals with defective proline catabolism [23–25, 29]. We next tested whether oolong tea extracts could also increase the shortened lifespan of *alh-6(lax105)* mutants. We found that exposure to oolong tea extracts significantly increased the mean lifespan of *alh-6(lax105)* worms when compared to the mock-treated control group (Fig. 1b, Fig. S1b, d, f, Table S1), and the increase in lifespan was more profound than wild-type worms treated with oolong tea extracts.

Because oolong tea extracts are comprised of a complex mixture of molecules, we next examined whether treatment with specific oolonghomobisflavans (OFs), found in *C. sinensis* oolong tea, could evoke similar increases in lifespan. Treatment with OFA or OFB resulted in similar increases in lifespan for both wild-type (Fig. 1c, Fig. S1g, i) and *alh-6(lax105)* (Fig. 1d, Fig. S1h, j) worms when compared to the mock-treated control group (Table S1). The increase in lifespan was similar to that observed in oolong tea extracts suggesting that OFs could be the active compounds in *C. sinensis* oolong tea extracts that improve the lifespan of *C. elegans*.

### Oolong tea extracts improve *C. elegans* healthspan

**Fig. 2:**
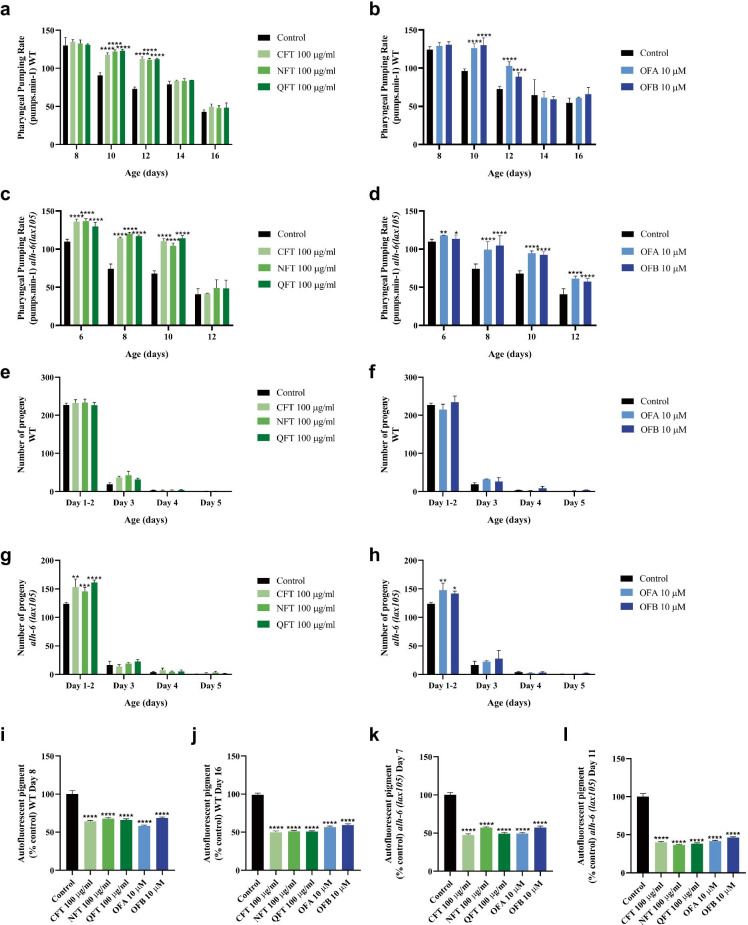
Effects of oolong tea on organismal health. Effect of oolong tea on pharyngeal pumping rate in wild-type (N2) (**a**, **b**) and *alh-6 (lax105)* (**c**, **d**) worms. The progeny output of wild-type (N2) (**e**, **f**) and *alh-6 (lax105)* (**g**, **h**). Effects of oolong tea on age-related marker (lipofuscin accumulation) in wild-type (N2) at day 8 (**i**) and day 16 (**j**) and *alh-6 (lax105)* at day 7 (**k**) and day 11 (**l**) of adulthood. Worms were treated with oolong tea (QFT, NFT, CFT, OFA, OFB) at 20 °C under standard laboratory conditions. *p < 0.05, **p < 0.01, ***p < 0.001, and ****p < 0.0001, compared to the untreated control by one-way ANOVA following Bonferroni’s method (post hoc)

Based on the observed increase in lifespan, we assessed multiple physiological functions over the lifespan of the worm, to investigate whether oolong tea extracts and OFs could also increase animal healthspan. We first measured the effect of oolong tea extracts and oolonghomobisflavans on pharyngeal pumping over the first three lifespan quartiles. Pharyngeal pumping rate is a well-established biomarker of aging, as they progressively decline with the animal’s age [47]. In wild-type animals, treatment with 100ug/ml QFT, NFT, or CFT delayed the age-related decline in pharyngeal pumping rate until day 12 of adulthood (Fig. 2a). Similarly, treatment of wild-type animals with OFA and OFB significantly delayed the age-associated reduction of pharyngeal pumping rates with age (Fig. 2b). The loss of mitochondrial proline catabolism leads to premature aging phenotypes, including lowered pharyngeal pumping rates [48]. This is reversed to wild-type levels when worms are treated with oolong tea extracts (Fig. 2c) or oolonghomobisflavans (Fig. 2d). Unlike wild-type animals, pharyngeal pumping in *alh-6(lax105)* worms treated with QFT, NFT, or CFT was only improved through day 10 of adulthood, while OFA and OFB treatment restored pharyngeal pumping to near wild-type levels at day 12 of adulthood. Importantly, oolong tea extracts and oolonghomobisflavan treatment did not affect the growth of the OP50 *E. coli* diet suggesting that the change in pharyngeal pumping rate is not due to an overt change in the composition of the bacterial diet, which can influence age-related physiology [23–25, 35] (Fig. S2a, b).

We next assessed whether treatment with oolong tea extracts and the oolonghomobisflavans (OFA and OFB) could influence *C. elegans* fertility. We measured total reproductive output and daily progeny production over the normal *C. elegans* reproductive span (Day 1–5 of adulthood) but observed no changes in wild-type animals treated with QFT, NFT, CFT (Fig. 2e, Fig. S2c) or treated with oolonghomobisflavans (Fig. 2f, Fig. S2c). In contrast, the reduction in progeny output of *alh-6(lax105)* mutant animals [48] was reversed when treated with oolong tea extracts (Fig. 2g, Fig. S2d) or oolonghomobisflavans (Fig. 2h, Fig. S2d).

In *C. elegans*, accumulation of intestinal lipofuscin, sometimes referred to as “age pigment”, accumulates with age and is an established biomarker of health or the rate of aging [47, 49]. Lipofuscin emits visible fluoresce following wavelength specific excitation and as such is quantifiable by microscopy. We examine the effects of QFT, NFT, CFT, OFA, and OFB treatment on intestinal autofluorescence at several points across the lifespan. In correlation with the improved lifespan, we measured a significant reduction of age pigment levels in wild-type animals that were treated as compared to the untreated control group on days 8 and 16 of adulthood (Fig. 2i, j, Fig. S2e, f). Similarly, treatment of *alh-6(lax105)* mutant worms reduced lipofuscin pigment accumulation at day 7 and day 11 of adulthood (Fig. 2k, l, Fig. S2g, h). Taken together, these data suggest that *C. sinensis* oolong tea extracts and oolonghomobisflavans can improve multiple health measures over the lifespan in wild-type animals and reverse the accelerate decline in multiple age-related phenotypes of animals with defects in mitochondrial proline catabolism.

### Oolong tea extracts promote stress resistance through DAF-16

**Fig. 3:**
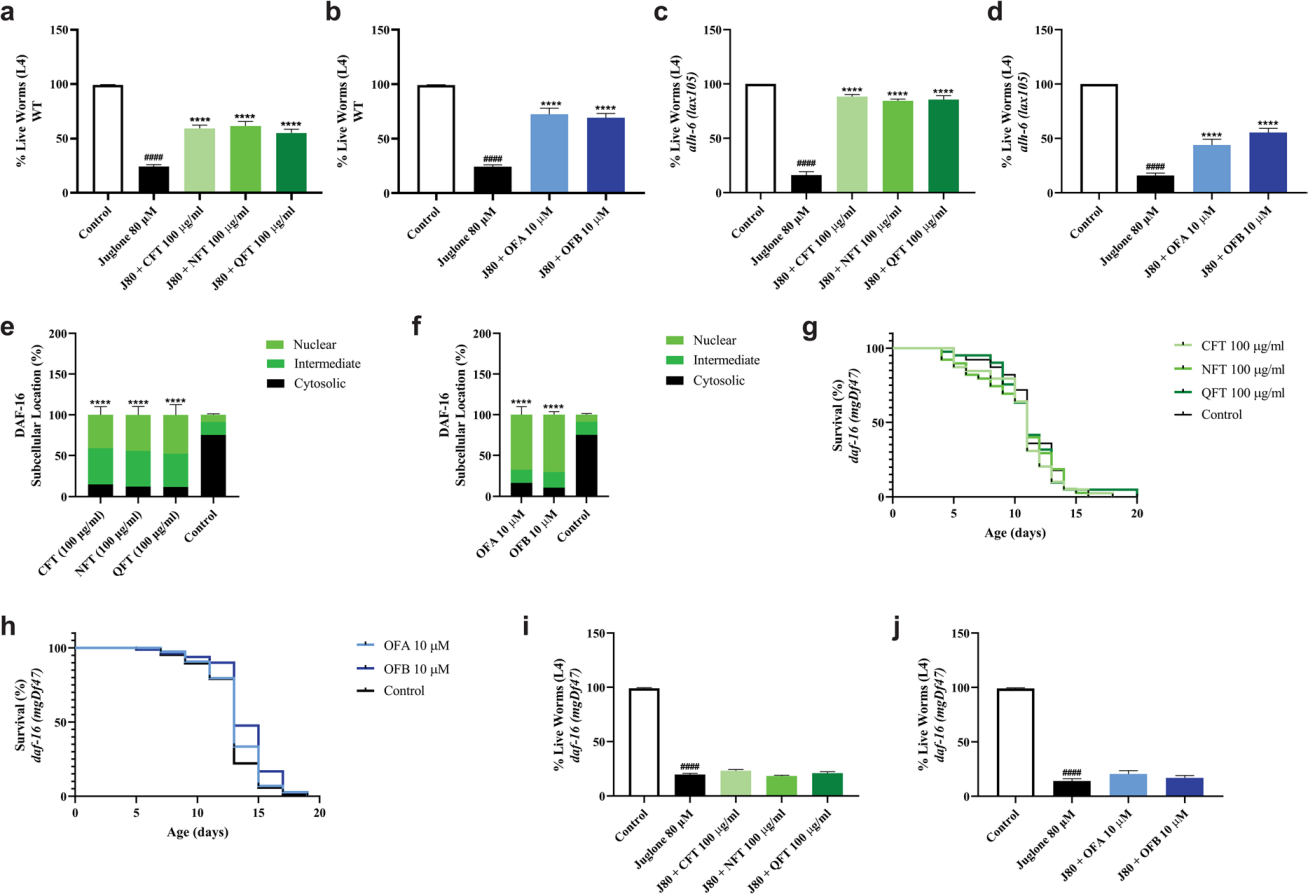
DAF-16 mediates the effects of oolong tea on stress resistance. The survival rate of wild-type (N2) (**a**, **b**) and *alh-6 (lax105)* (**c**, **d**) worms under juglone-induced oxidative stress conditions. The subcellular distribution of DAF-16::GFP (**e**, **f**). Effects of oolong tea on stress resistance and longevity of *daf-16 (mgDf47)* mutant worms; survival curves of *daf-16 (mgDf47)* mutant worms under normal conditions (**g**, **h**) and survival rate of *daf-16 (mgDf47)* mutant worms under juglone-induced oxidative stress conditions (**i**, **j**). Worms were treated with oolong tea (QFT, NFT, CFT, OFA, OFB) at 20 °C under standard laboratory conditions. ^####^*p* < 0.0001 compared to the untreated control. *p < 0.05, **p < 0.01, ***p < 0.001, and ****p < 0.0001, compared to the juglone treated group by one-way ANOVA following Bonferroni’s method (post hoc)

With age comes a decline in the capacity of an organism to tolerate exposure to extreme environmental stress conditions [50]. Based on the impact that *C. sinensis* extracts and oolonghomobisflavans have on *alh-6(lax105)* mutant lifespan and healthspan, we predicted that QFT, NFT, CFT, OFA, and OFB would enhance organismal survival under oxidative stress conditions. In support of this hypothesis, we found that treatment with these compounds significantly increased the survival rate of both wild-type (Fig. 3a, b) and *alh-6(lax105)* (Fig. 3c, d) worms under juglone-induced oxidative stress conditions; similar results were observed when animals were treated with vitamin C and N-acetylcysteine (Fig. S3a, b). These data suggest that QFT, NFT, CFT, OFA, and OFB exhibit antioxidant-like properties.

We next investigated the molecular genetic pathways that mediate the effects from oolong tea extracts and oolonghomobisflavans on *C. elegans* lifespan and healthspan. DAF-16 is a FOXO-family transcription factor with well-established roles in mediating longevity and stress resistance [51, 52]. In response to changes in cellular homeostasis, the subcellular localization of DAF-16 is shifted toward the nucleus where it activates genes required for stress adaptation [53]. We first examined the effects of *C. sinensis* extracts and oolonghomobisflavans on the nuclear/cytoplasmic dynamics of DAF-16 using a DAF-16::GFP reporter strain [54]. When compared to the untreated control group, worms treated with oolong tea extracts displayed increased nuclear location of DAF-16::GFP (Fig. 3e, Fig. S3c–f). A similar increase in DAF-16::GFP nuclear location was observed following treatment with OFA and OFB (Fig. 3f, Fig. S3c–f). Moreover, DAF-16 transcriptional activity was enhanced as the DAF-16 target genes *sod-2*, *sod-3, sod-4,* and *dod-17* [55] were upregulated after OFA and OFB treatment (Fig. S3g).

To investigate whether *daf-16* is required for the enhancement of lifespan and healthspan by oolong tea extracts and oolonghomobisflavans, we treated *daf-16(mgDf47)* mutant animals with QFT, NFT, CFT, OFA, and OFB and measured the effect on lifespan and oxidative stress resistance. We did not observe an increase in lifespan of *daf-16(mgDf47)* animals treated with any oolong tea extract (Fig. 3g) or oolonghomobisflavans (Fig. 3h), and neither treatment was able to increase *daf-16(mgDf47)* survival rate under oxidative stress (Fig. 3i, j). Taken together, these data reveal that DAF-16 is required for the lifespan and healthspan promoting effects of oolong tea extracts and oolonghomobisflavans.

### Oolonghomobisflavan A and oolonghomobisflavan B are neuroprotective

Oxidative stress can accelerate age-related pathologies and has been extensively studied in the progression of neurodegenerative diseases [5]. Previous work has demonstrated that the oolong tea extracts QFT, NFT, CFT can protect against Aβ-induced neurotoxicity in *C. elegans* [56]; however, the oolonghomobisflavans have not been tested. In light of the similar antioxidant-like effects of oolonghomobisflavans as compared to QFT, NFT, and CFT, we examined whether treatment of *C. elegans* with oolonghomobisflavans A or oolonghomobisflavans B was neuroprotective.

**Fig. 4:**
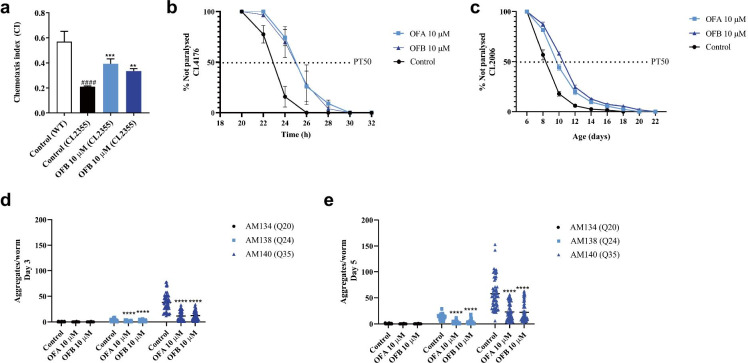
OFA and OFB are neuroprotective and delay protein aggregation. Neuroprotective effect of OFA and OFB against Aβ-induced toxicity; the Aβ-induced chemotactic dysfunction index of CL2355 mutant worms (**a**), the Aβ-induced paralysis of CL4176 (**b**), and CL2006 mutant worms (**c**). Proteostasis effects of OFA and OFB against polyQ-induced toxicity; the polyQ accumulation of AM134 (Q20), AM138 (Q24), and AM140 (Q35) mutant worms at day 3 (**d**) and day 5 (**e)** of adulthood. ^####^*p* < 0.0001 for comparison to the untreated control (wild-type (N2) worms). *p < 0.05, **p < 0.01, ***p < 0.001, and ****p < 0.0001, compared to the untreated control by one-way ANOVA following Bonferroni’s method (post hoc)

We explored the effects of oolong tea extracts and oolonghomobisflavans on Aβ-induced neurotoxicity using transgenic animals expressing human Aβ1–42 in the neuronal cells (pan-neuronal) that drive progressive defects in chemotactic behavior toward the volatile attractant diacetyl (Fig. 4a) [57]. Treatment with oolonghomobisflavans A or oolonghomobisflavans B partially restored chemotaxis toward diacetyl, indicating an improvement in neuronal function (Fig. 4a, Fig. S4a). This restoration of chemotactic response was similar in animals treated with to QFT, NFT, and CFT (Fig. S4b) as previously reported [56]. We found that oolong tea extracts, OFA, and OFB did not affect the chemotaxis behavior of the control transgenic worms without neuronal Aβ expression (Fig. S4a, c, d), which supports a role in neuronal protection from stress rather than enhancement of normal activity.

### Oolonghomobisflavan A and oolonghomobisflavan B improve proteostasis

Expression of human Aβ1–42 results in the formation of cytotoxic protein aggregates [57, 58]. To better define the effects of oolonghomobisflavans on Aβ proteostasis we utilized a strain that expresses Aβ1–42 in the musculature of *C. elegans*, which displays Aβ aggregation-dependent paralysis [58], and can be more sensitive than Aβ-dependent effects on chemotaxis. We employed two different transgenic strains that display either temperature-dependent (*myo-3p::Aβ1–42; smg-1*) or age-dependent (*unc-54p::Aβ1–42*) paralysis and found that treatment with oolonghomobisflavan A and oolonghomobisflavan B resulted in a clear delay in the time it takes for 50% of the worms to become paralyzed (PT50) as compared to the untreated control group, for both the temperature-dependent (Fig. 4b, Fig. S4e) and age-dependent (Fig. 4c, Fig. S4f) paralysis models.

To examine whether oolonghomobisflavan A and oolonghomobisflavan B could impact other models of proteotoxicity, we subsequently tested polyglutamine (polyQ) models of protein aggregation in the *C. elegans* muscle. Strikingly, treatment with oolonghomobisflavan A and oolonghomobisflavan B reduced protein aggregation in animals expressing YFP with 24-polyQ repeats (Q24) or YFP with 35-polyQ repeats (Q35) in the muscle at day 3 and day 5 of adulthood (Fig. 4d, e, Fig. S4g); no significant change was observed in a control strain with YFP (without polyQ repeats, Q20) in muscle treated with either oolonghomobisflavan. Taken together, oolong tea has potentially protective effects against Aβ- and polyQ-induced toxicity which might alleviate protein misfolding and maintain protein homeostasis.

## Discussion

Tea polyphenols have gained popularity in research because of their antioxidant properties and potential for health benefits. Most prominently, the polyphenolic compound EGCG in green tea and theaflavins in black tea has been reported to potentially oppose age-related conditions [36, 59–61]. The health-promoting effects of tea extracts is a physiological response that has been demonstrated in *Drosophila melanogaster* [59, 61] and rodents [62]. Although tea extracts exhibited potential effects on longevity, EGCG alone could not extend the lifespan of *C. elegans* [63], which suggested that additional bioactive molecules in tea extracts must be responsible for the health and longevity effects. In the present study, we demonstrate the health benefits and longevity effects of *C. sinensis* oolong tea and its extracted compounds oolonghomobisflavan A and oolonghomobisflavan B in *C. elegans*.

Tieguanyin is one variety of the Chinese oolong tea (*C. sinensis*) [44]. In this study the different types of oolong tea used included Qing flavor Tieguanyin oolong tea (QFT), Nong flavor Tieguanyin oolong tea (NFT), and Chen flavor Tieguanyin oolong tea (CFT), which differ in the degree of fermentation. QFT, NFT, and CFT were fermented for 1 year (%), 2 years (%), and 10 years (%), respectively. During tea processing, extended fermentation times can reduce the effective concentrations of polyphenol content while increasing the caffeine content [43]. Surprisingly, the length of fermentation had little effect on the polyphenol contents of CFT, NFT, and QFT [56].

Our results expand upon the previous observations that complex mixtures of compounds from tea extracts can increase lifespan, and we define OFA and OFB as potent molecules with lifespan and healthspan promoting characteristics. Moreover, our studies define the healthspan enhancing potential of oolong tea extracts, OFA and OFB, thus demonstrating that oolong tea extracts and specifically OFA and OFB can prolong lifespan and improved healthspan without significant damage to physiological function.

Previous studies have suggested that tea extract can help maintain oxidative homeostasis [36, 37]. Aging and related diseases are closely associated with oxidative stress [3]. The lifespan extension of worms was closely related to stress resistance properties [50]. Oolong tea improved stress resistance and effectively mounts a molecular response to decrease reactive oxygen species, suggesting that its oxidative stress resistance properties partially contributed to its pro-longevity activity.

Our results support this idea and provide additional evidence that oolong tea can attenuate oxidative stress through the modulation of the stress-response gene expression pathways including the insulin signaling pathway (IIS). Specifically, the beneficial effects of oolong tea extracts, OFA, and OFB on lifespan and stress resistance were lost in animals lacking a functional allele of the IIS regulated transcription factor DAF-16. Our results add OFA and OFB to a collection of naturally-derived polyphenols which have been previously reported to exhibit health-promoting activities, particularly through antioxidant properties; including EGCG [20, 64], epicatechin [20, 65], anthocyanin [45], resveratrol [2, 18], and quercetin [66, 67].

Previously, Wu et al. reported that the combination of tea polyphenols and tea polysaccharides (rhamnose, glucose, galactose, arabinose, and xylose) or crude extracts of oolong tea have more beneficial effects in preventing obesity in mice than tea polyphenols or tea polysaccharides alone [68]. Moreover, Xu et al. reported that there have synergistic effects of tea polyphenols and polysaccharides in anti-inflammatory activity [42]. Based on our observations in this study, the health benefit of oolong tea extracts can be attributed, at least in part, to the effects of OFA and OFB.

Mitochondria are essential organelles with a complex relationship between health and longevity. Mitochondria are a major source of cellular ROS that influence the aging process [69]. We further confirmed the health benefits of oolong tea in the *alh-6(lax105)* proline catabolism pathway mutant model that displays age-dependent mitochondrial dysfunction. Previously, we demonstrated that *alh-6(lax105)* loss-of-function worms exhibit increased ROS generation, altered mitochondrial homeostasis, and shorten lifespan (without development delay). Strikingly, the health-promoting effects of tea extracts, OFA, and OFB, could overcome the oxidative stress induced by loss of the mitochondrial proline catabolism pathway gene *alh-6* [48] including shortened lifespan and premature health deterioration. We noted that the enhanced effects tea extracts, which likely result from the diversity of antioxidants found in tea extracts beyond OFA and OFB [70, 71]. Future studies to define the molecular mechanisms underlying the relationship between oolong tea extracts, OFA, and OFB on mitochondria homeostasis will be of critical importance.

## Methods

### *C. elegans* strains and maintenance

All strains were cultured on nematode growth media (NGM) supplemented with *Escherichia coli* OP50 using standard methods [72]. Worms were maintained at 20 °C, unless otherwise noted. Strains used in this study include N2 Bristol wild-type (N2), TJ356 (*zIs356 [daf-16p::daf-16a/b::GFP* + *rol-6]*), *daf-16 (mgDf47)*, CL4176 (*smg-1(cc546) I; dvIs27 [(myo-3p::A-Beta (1–42)::let-851 3′UTR)* + *rol-6(su1006)] X*), CL2006 (*dvIs2 [pCL12(unc-54/human Abeta peptide 1–42 minigene)* + *pRF4]*), CL2355 (*smg-1(cc546) dvIs50 [pCL45 (snb-1::Abeta 1–42::3′ UTR(long)* + *mtl-2::GFP] I*), CL2122 (*dvIs15 [(pPD30.38) unc-54(vector)* + *(pCL26) mtl-2::GFP]*), AM134 (*rmIs126 [unc-54p::Q20::YFP]*), AM138 (*rmIs126 [unc-54p::Q24::YFP]*), AM140 (*rmIs126 [unc-54p::Q35::YFP]*), SPC223 *alh-6(lax105);*[*gst-4p::gfp*].

For some experiments, as noted, worms were grown in liquid S-medium which was prepared by mixing with *E. coli* OP50 (OD600 = 1.0). Age-synchronized populations of worms were obtained by hypochlorite treatment [73].

### Tea preparation

Dried oolong tea leaves (30 g) were purchased from Taoyuan Organic Tea (Anxi County, Fujian Province, China) and extracted with distilled water as described previously [56]. The leaves were infused twice with boiled distilled water (1 L; 100 °C) for 30 min. The combinations of extracts from two rounds were filtered using Whatman no. 1 filter paper, followed by lyophilization. Oolong tea extracts were stored at − 20 °C and dissolved in distilled water before use. Oolonghomobisflavan A (OFA) (CAS No. 126737-60-8, Cat No. NS240102) and oolonghomobisflavan B (OFB) (CAS No. 176107-91-8, Cat No. NS240202) were purchased from Nagara Science Co. (Gifu, Japan).

### Lifespan assay

Worms were synchronized to generate a synchronous L1 population. Larval stage 4 (L4) worms were moved to NGM agar plates supplemented with oolong tea extracts or OFs. The different concentrations of oolong tea extracts were prepared in M9 buffer and placed above *E. coli* OP50 lawn and incubated at room temperature overnight before use. Animals were observed and moved to fresh medium every second day until the end of life. Worms that failed to respond to a gentle touch were scored as dead. Animals with internally hatched progeny, extruded gonads, or crawled to the side of the plate were censored. Each experimental replicate measured a minimum of 30 individual animals for a total of 90–120 animals/treatment.

### Pharyngeal pumping assay

Pharyngeal pumping assays and lifespan assays were conducted at the same time. On the 8th, 10th, 12th, 14th, and 16th day of adulthood for wild-type (N2) worms and the 6th, 8th, 10th, and 12th day of adulthood in *alh-6 (lax105)* worms. The pharyngeal pumping rates were quantified by counting pharynx contractions for 60 s. Each experimental replicate measured a minimum of 20 individual animals for a total of 60–90 animals/treatment.

### Age pigment fluorescence (lipofuscin) assay

WT and *alh-6(lax105)* animals at L4 larval stage were treated with different concentrations of oolong tea extracts or OFs in S-medium for 7, 8, 11, and 16 days. The media was changed every second day. After treatment, worms were anesthetized with 10 mM tetramisole and subsequently transferred on a microscopic glass slide (supplemented with a 1% agarose pad). The fluorescence images were acquired using ZEN software and Zeiss Axio Imager (λex 360/20 nm, λem 460/38 nm). The intensity of fluorescence was analyzed using Fiji ImageJ-win64 (Max Planck Institute of Molecular Cell Biology and Genetics, Dresden, Germany).

### Reproduction assays

WT and *alh-6(lax105)* worms were synchronized in the same way as in the lifespan assay.

The L4 larval stage animals were sorted and placed one by one on each NGM agar plate supplemented with oolong tea extracts. For brood size assays, L4 worms were singled on NGM agar plate supplemented with oolong tea extracts and incubated at 20 °C for 24 h. Each group had a minimum of 20 worms. The adult worms were moved every 12 h until egg-laying ceased. The eggs were counted using a dissecting microscope every day for 5 days to obtain a number of progeny and a mean brood size.

### Survival assay

WT, *alh-6(lax105)*, and *daf-16(mgDf47)* worms at L1 larval stage were treated with different concentrations of oolong tea extracts in S-medium for 48 h. Each group was further treated with 80 μM pro-oxidant juglone for 24 h. The survivors were counted after 24 h of juglone treatment.

### DAF-16 subcellular localization

The TJ356 transgenic strain stably expresses a DAF-16::GFP fusion protein. TJ356 transgenic worms at L1 larval stage were treated with different concentrations of oolong tea extracts or OFs in S-medium for 24 h. After incubation, worms were submitted to Zeiss Axio Imager microscopy as described before. Subsequently, the DAF-16 translocation in TJ356 worms was classified into three categories including cytosolic, intermediate, and nuclear (Fig. S3c–e).

### Quantitative RT-PCR

RNA extraction was performed with TRI reagent. Quantitative real-time PCR was performed using standard procedures [35, 74–76]. The primer sequences are listed in the Supplementary Materials (Table S2). Expression was normalized to *snb-1* values [77].

### Paralysis assay

The CL4176 and CL2006 transgenic strain express human Aβ1–42 in muscle cells, as described previously [57, 58]. The transgenic worms were synchronized and treated with oolong tea extracts or OFs at the L4 stage on NGM agar plate. After treatment, CL4176 transgenic worms were maintained at 15 °C for 48 h and moved to 25 °C to induce Aβ expression. The number of paralyzed worms was counted at 20, 22, 24, 26, 28, and 30 h after moving to 25 °C. The CL2006 worms were maintained at 20 °C. Worms were classified as paralyzed when they did not move or only moved their head (cleared bacteria giving a halo appearance around the worms’ heads). Paralyzed worms were recorded and excluded from the plates every second day.

### Chemotaxis assay

The CL2122 and CL2355 transgenic strains express human Aβ1–42 in neuronal cells, as described previously [57]. The transgenic worms were synchronized and treated with oolong tea extracts or OFs at the L4 stage on NGM agar plate. After treatment, CL2122 and CL2355 transgenic worms were maintained at 15 °C for 36 h and moved to 25 °C for 36 h to induce Aβ1–42 expression. The worms were washed with M9 buffer and placed in the center of the chemotaxis plate. The chemotaxis plates (20 mm, NGM agar, without antibiotic) were prepared before the experiment as followed: the attractant side contained a mixture of diacetyl (0.1% in absolute ethanol) and sodium azide (0.5 M) on the plate, the control side (opposite side of the plate) contained a mixture of absolute ethanol and sodium azide (0.5 M). After the placement, worms were allowed to stay in 25 °C for 1 h before recording the number of worms at each location. The chemotaxis index (CI) was defined as follows: ((the number of worms at attractant side—the number of worms at the control side)/the total number of worms).

### PolyQ aggregation assay

The mutant strains AM134 (Q20), AM138 (Q24), and AM140 (Q35) were used for polyQ aggregation assay [78]. The AM138 and AM140 strain express a Q24 and Q35 region fused to YFP in the muscle cells, resulting in an appearance of fluorescent spots in the body wall of muscle cells during aggregation [79].

The AM transgenic worms at L1 larval stage were treated with different concentrations of oolong tea extracts or OFs in S-medium. The media was changed every second day. On day 3 and day 5 of adulthood, worms were anesthetized and submitted to Zeiss Axio Imager microscopy as described before. The number of polyQ::YFP aggregates in body wall muscle was counted. Approximately 30 nematodes were randomly selected in each treatment group and scored for aggregates.

### Statistical analysis

Data are presented as mean ± SEM (n, indicated for each experiment, replicated a minimum of three times). Data were analyzed by one-way ANOVA following Bonferroni’s method (post hoc). Data handling and statistical processing were performed using GraphPad Prism 8.0. Differences were considered significant at the *p* ≤ 0.05 level.

## Supplementary Information

Below is the link to the electronic supplementary material.

Supplementary file1 (DOCX 992 kb)

## Data Availability

The data that supports the findings of this study are available in the supplementary material of this article.
