# Supplementary material for: Oolonghomobisflavans from Camellia sinensis increase Caenorhabditis elegans lifespan and healthspan
Source: GeroScience. 2021 Oct 12;44(1):533–45. doi: 10.1007/s11357-021-00462-7 (PMC8811050; doi:10.1007/s11357-021-00462-7)
Supplement: Supplementary file 1 — Supplementary file1 (DOCX 992 kb) [file 11357_2021_462_MOESM1_ESM.docx]

**SUPPLEMENTARY DATA**

**Supplementary Table 1.** **Results and statistical analyses of oolong tea treated *C. elegans* in lifespan assay.** Statistics of lifespan data from Figure 1 and S1.

| Strain | Condition | Maximum survival  (days) | Mean survival  (days) | % mean survival vs. control | *P* value  (log-rank) treatment vs. control | Combined  Number of animals  (N=3) | Number Censored |
| --- | --- | --- | --- | --- | --- | --- | --- |
| Wild-type  (N2) |  |  |  |  |  |  |  |
|  | Control | 24 | 15.56±0.28 |  |  | 86 | 7 |
|  | CFT 50 μg/ml | 26 | 16.89±0.30 | 8.55 | < 0.0001 | 81 | 11 |
|  | CFT 100 μg/ml | 26 | 17.36±0.34 | 11.57 | < 0.0001 | 91 | 8 |
|  | CFT 200 μg/ml | 26 | 18.21±0.39 | 17.03 | < 0.0001 | 83 | 6 |
|  | NFT 50 μg/ml | 26 | 16.67±0.38 | 7.13 | < 0.0001 | 83 | 10 |
|  | NFT 100 μg/ml | 26 | 17.47±0.53 | 12.28 | < 0.0001 | 73 | 10 |
|  | NFT 200 μg/ml | 24 | 17.03±0.32 | 9.45 | < 0.01 | 78 | 7 |
|  | QFT 50 μg/ml | 24 | 17.42±0.36 | 11.95 | < 0.0001 | 91 | 12 |
|  | QFT 100 μg/ml | 26 | 17.09±0.37 | 9.83 | < 0.001 | 81 | 6 |
|  | QFT 200 μg/ml | 26 | 17.78±0.36 | 14.27 | < 0.0001 | 99 | 11 |
|  | OFA 5 μM | 26 | 17.10±0.20 | 9.90 | < 0.0001 | 103 | 7 |
|  | OFA 10 μM | 28 | 17.65±0.22 | 13.43 | < 0.0001 | 116 | 4 |
|  | OFA 25 μM | 32 | 18.42±0.28 | 18.38 | < 0.0001 | 101 | 10 |
|  | OFB 5 μM | 34 | 18.52±0.32 | 19.02 | < 0.0001 | 102 | 7 |
|  | OFB 10 μM | 32 | 17.08±0.23 | 9.77 | < 0.0001 | 106 | 12 |
|  | OFB 25 μM | 34 | 17.10±0.25 | 9.90 | < 0.0001 | 95 | 7 |
| *alh-6 (lax105)* |  |  |  |  |  |  |  |
|  | Control | 19 | 11.12±0.29 |  |  | 84 | 6 |
|  | CFT 50 μg/ml | 27 | 14.54±0.35 | 30.76 | < 0.0001 | 96 | 3 |
|  | CFT 100 μg/ml | 25 | 14.33±0.47 | 28.87 | < 0.0001 | 99 | 8 |
|  | CFT 200 μg/ml | 25 | 15.30±0.52 | 37.59 | < 0.0001 | 98 | 8 |
|  | NFT 50 μg/ml | 27 | 14.91±0.49 | 34.08 | < 0.0001 | 107 | 3 |
|  | NFT 100 μg/ml | 27 | 15.84±0.51 | 42.45 | < 0.0001 | 110 | 3 |
|  | NFT 200 μg/ml | 27 | 15.95±0.58 | 43.44 | < 0.0001 | 96 | 3 |
|  | QFT 50 μg/ml | 29 | 15.50±0.52 | 39.39 | < 0.0001 | 112 | 2 |
|  | QFT 100 μg/ml | 25 | 15.41±0.49 | 38.58 | < 0.0001 | 102 | 2 |
|  | QFT 200 μg/ml | 31 | 16.38±0.59 | 47.30 | < 0.0001 | 113 | 6 |
|  | OFA 5 μM | 25 | 16.44±0.60 | 47.84 | < 0.0001 | 70 | 8 |
|  | OFA 10 μM | 31 | 17.49±0.59 | 57.28 | < 0.0001 | 69 | 6 |
|  | OFA 25 μM | 33 | 16.53±0.65 | 48.65 | < 0.0001 | 74 | 5 |
|  | OFB 5 μM | 27 | 18.41±0.60 | 65.56 | < 0.0001 | 64 | 7 |
|  | OFB 10 μM | 25 | 16.18±0.69 | 45.50 | < 0.0001 | 54 | 5 |
|  | OFB 25 μM | 29 | 15.27±0.61 | 37.32 | < 0.01 | 57 | 3 |

**Table S2. Primer sequences for *C. elegans* candidate genes.**

| Gene  Target | Primer | Sequence |
| --- | --- | --- |
| *sod-2* | F | 5’-GCTCTTCAGCCAGCTCTCAA-3’ |
|  | R | 5’-TGGTTCTCCTCCGTCCTTTG-3’ |
| *sod-3* | F | 5’-GGGAGCACGCCTACTACTTG-3’ |
|  | R | 5’-AGCATTGGCAAATCTCTCGC-3’ |
| *sod-4* | F | 5’-TGCACCAGATGACTCGAACA-3’ |
|  | R | 5’-TGAGGCAAGAGAGTCGGAAAC-3’ |
| *dod-17* | F | 5’-GTGGATGCTGCAGGAAATCT-3’ |
|  | R | 5’-GTTGGAAGTGGCCACCAAAC-3’ |
| *snb-1* | F | 5’-CCGGATAAGACCATCTTGACG-3’ |
|  | R | 5’-GACGACTTCATCAACCTGAGC-3’ |

**
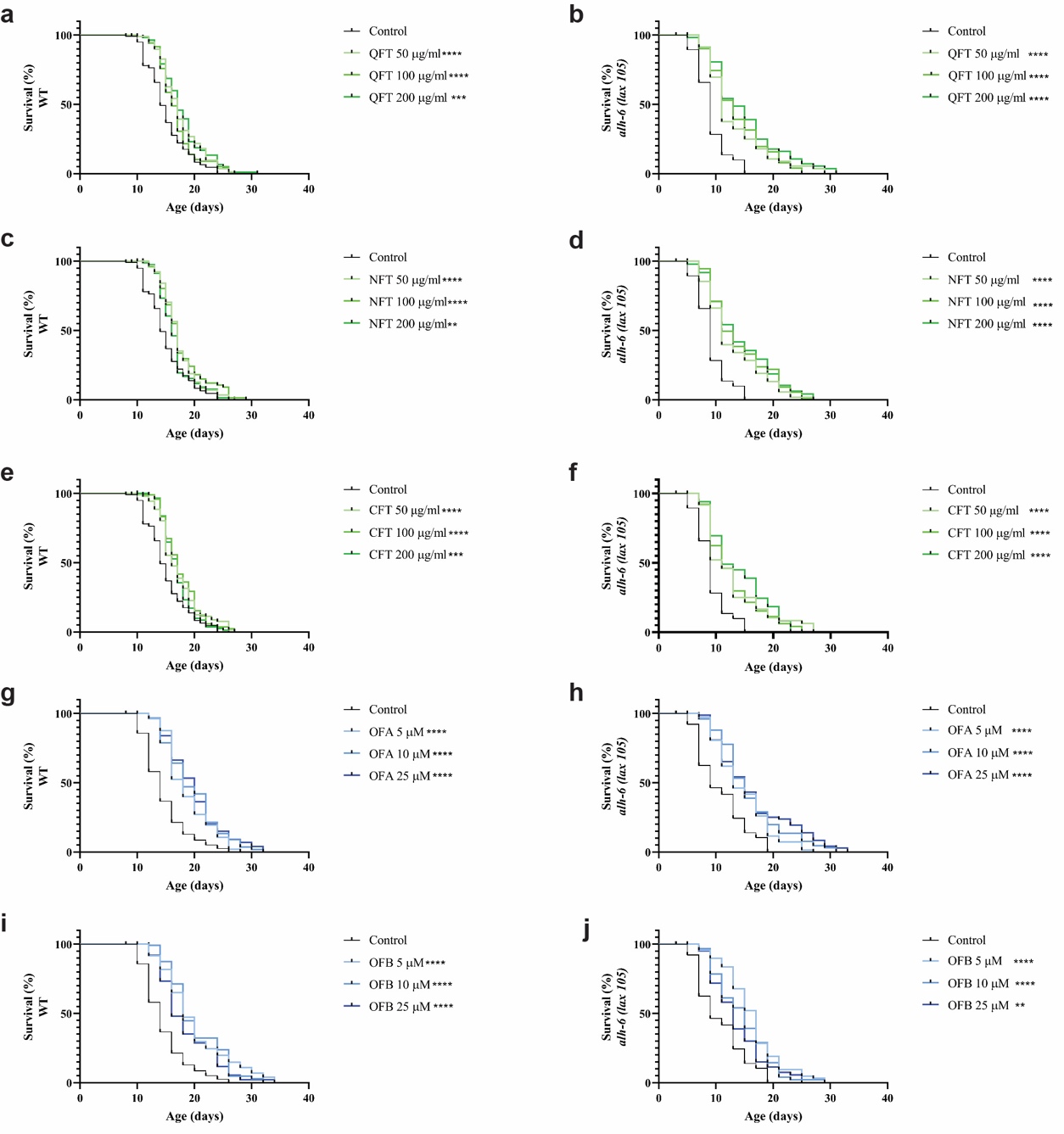
**

**Figure S1. Establishing the effective concentration of oolong tea extracts on lifespan extension.** Survival curves of wild-type (N2) and *alh-6(lax105)* worms at 20 °C on the plate treated with different concentrations of QFT **(a,b)**, NFT **(c,d)**, CFT **(e,f)**, OFA **(g,h)**, OFB **(i,j)**. **p*<0.05, ***p*<0.01, ****p*<0.001 and *****p*<0.0001, compared to the mock-treated control by one-way ANOVA following log-rank test.


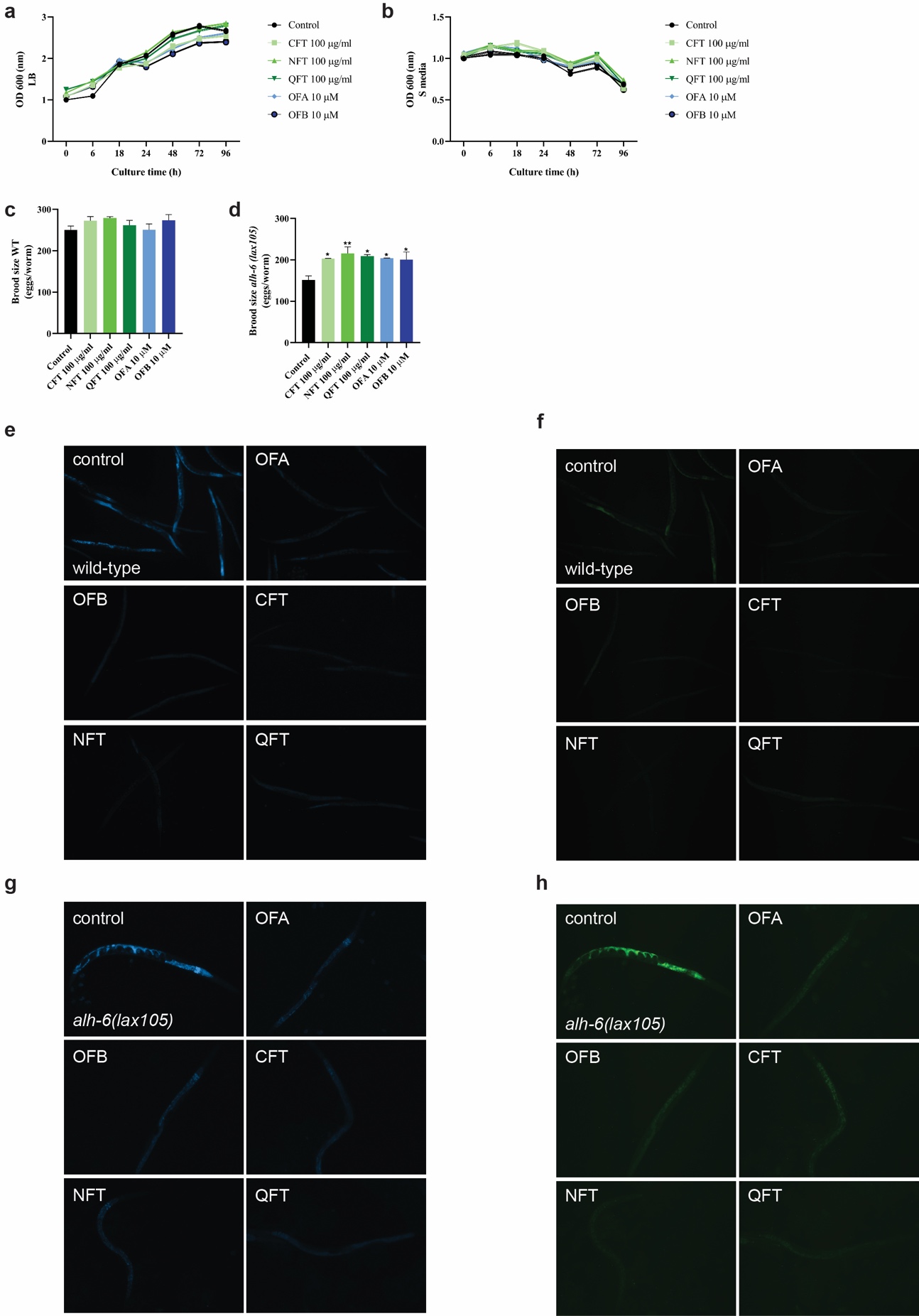


**Figure S2. The effects of oolong tea on *E. coli* OP50 growth**. OP50 was inoculated in LB **(a)** and S-media **(b)** and growth measured at indicated timepoints. The total brood size of wild-type (N2) **(c)** and *alh-6 (lax105)* **(d)** worms after treatment with oolong tea (QFT, NFT, CFT, OFA, OFB). **p*<0.05, ***p*<0.01, ****p*<0.001 and *****p*<0.0001, compared to the untreated control by one-way ANOVA following Bonferroni’s method (post hoc). Representative pictures of lipofuscin accumulation of wild-type (N2) worms after treated with oolong tea (QFT, NFT, CFT, OFA, OFB) until day 8 of adulthood **(e;** blue channel**) (f;** green channel**).** Representative pictures of lipofuscin accumulation of *alh-6 (lax105)* worms after treated with oolong tea (QFT, NFT, CFT, OFA, OFB) until day 7 of adulthood **(g;** blue channel**) (h;** green channel**).**

**
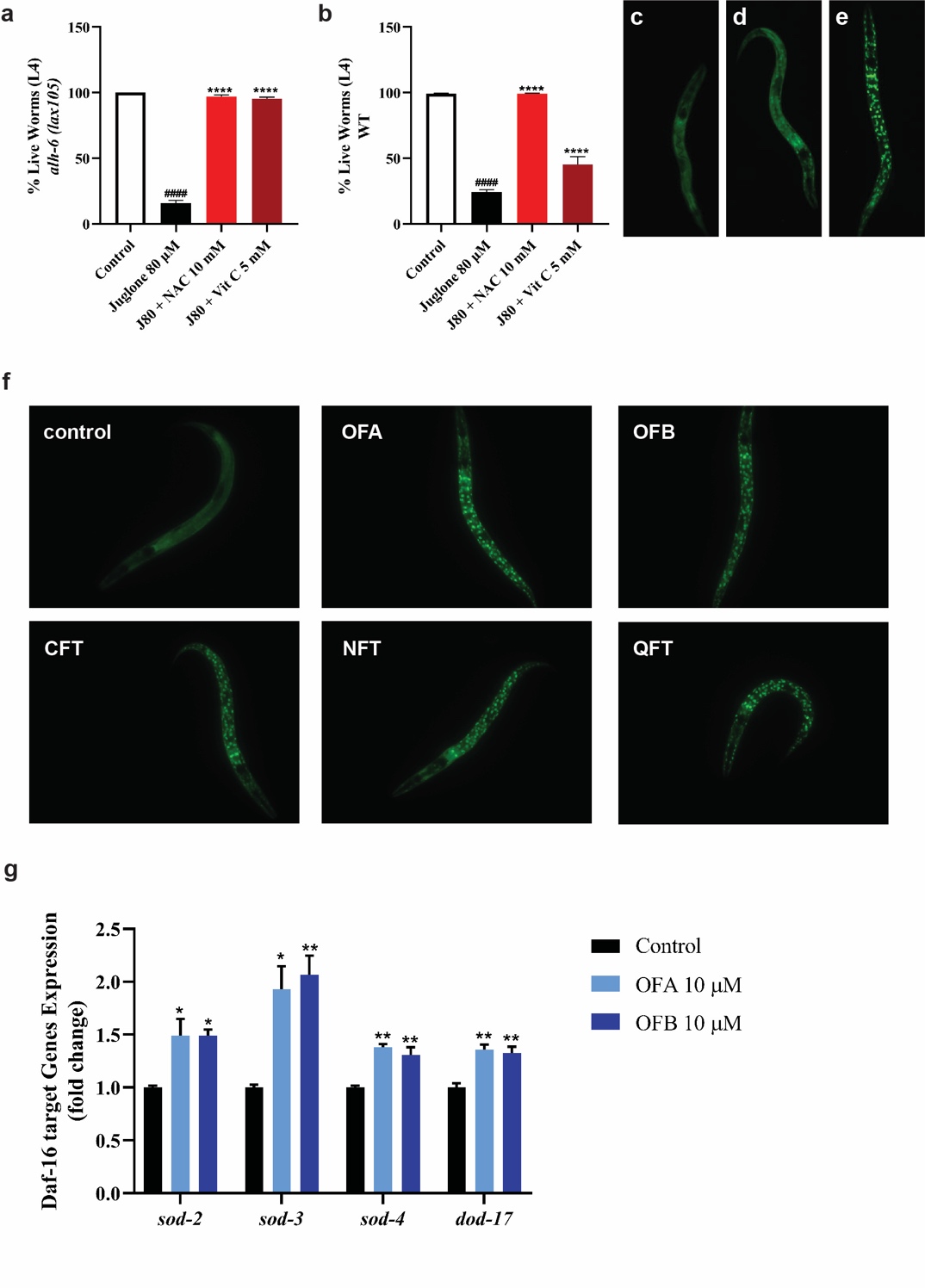
**

**Figure S3. Antioxidant effects of OFA and OFB.** The survival rate of wild-type (N2) and *alh-6 (lax105)* worms under juglone-induced oxidative stress conditions after incubation with NAC (10 mM) **(a)** or Vit C (5mM) **(b)**. Representative fluorescent pictures of the subcellular location of DAF-16 in the nuclear **(c)**, intermediate **(d)** and cytosolic regions **(e)**. Representative pictures of DAF-16::GFP in TJ356 mutant worms after treated with oolong tea (QFT, NFT, CFT, OFA, OFB) **(f)**. (**g**). Effect of OFA and OFB on DAF-16 target genes expression. Expression levels normalized to the untreated control. **p*<0.05, ***p*<0.01, ****p*<0.001 and *****p*<0.0001, ^####^ *p*<0.0001 for comparison to the untreated control by one-way ANOVA following Bonferroni’s method (post hoc). **p*<0.05, ***p*<0.01, ****p*<0.001 and *****p*<0.0001, compare to the juglone treated group by one-way ANOVA following Bonferroni’s method (post hoc).

**
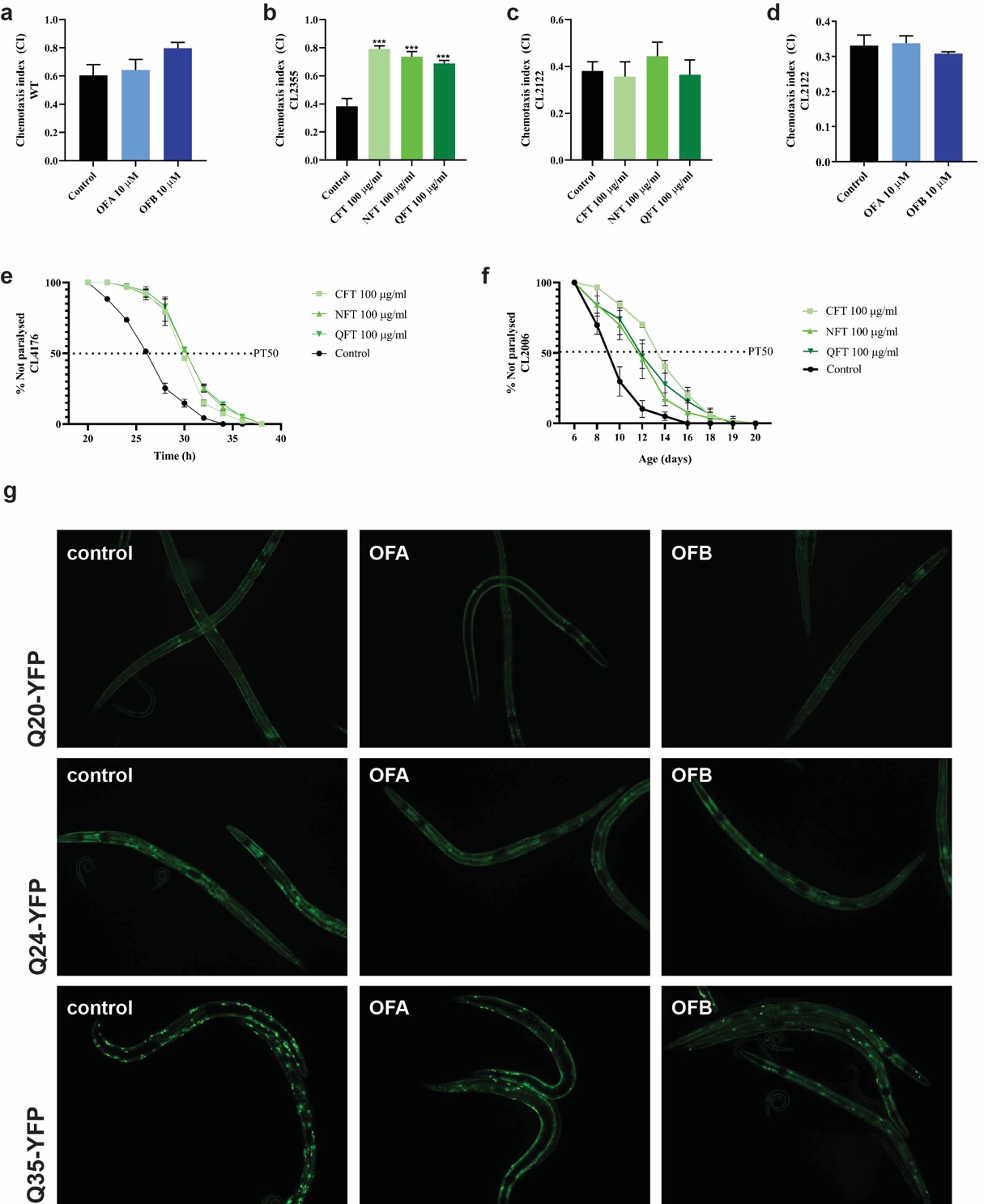
**

**Figure S4. Effects of oolong tea on neuroprotection.** The chemotactic dysfunction index of wild-type (N2) worms after treatment with OFA and OFB **(a).** The chemotactic dysfunction index of CL2355 mutant worms after treatment with oolong tea extracts (QFT, NFT, CFT) **(b)**. The chemotactic dysfunction index after treatment of transgenic control strain (CL2122) with oolong tea (QFT, NFT, CFT, OFA, OFB) **(c,d).** Paralysis curves of CL4176 **(e)** and CL2006 **(f)** mutant worms after treated with oolong tea extracts (QFT, NFT and CFT). Representative pictures of polyQ aggregation in AM134 (Q20), AM138 (Q24), AM140 (Q35) mutant worms at day 5 **(g)** of adulthood after treated with OFA and OFB.
